# Neuro-Transcriptomic Responses to Polypharmacological Agents in *Danio rerio*: Implications for Translational Drug Repurposing in Neurodevelopmental Disorders

**DOI:** 10.3390/brainsci16030323

**Published:** 2026-03-18

**Authors:** Alexander D. Bartkowiak, Marie R. Mooney

**Affiliations:** 1 Department of Pharmacology and Therapeutics, College of Medicine, University of Florida, Gainesville, FL 32610, USA; abartkowiak@ufl.edu; 2 Department of Biology, College of Arts and Sciences, University of North Florida, Jacksonville, FL 32224, USA; 3 Stanley Manne Children’s Research Institute, Louis A. Simpson and Kimberly K. Querrey Biomedical Research Center, Chicago, IL 60611, USA; 4 Feinberg School of Medicine, Northwestern University, Chicago, IL 60611, USA

**Keywords:** neurodevelopmental disorders, drug repurposing, transcriptomics, zebrafish, GABA

## Abstract

**Highlights:**

**What are the main findings?**

- This method rapidly extracts transcriptional signatures directly from intact primary neurons.
- Distinct GABA-acting drugs produce shared transcriptional signatures in zebrafish neurons influencing both GABAergic and glutamatergic synapses.

**What are the implications of the main findings?**

- A focus on rapid-omics in neurons can accelerate repurposing efforts for neuroactive compounds with differential affinities for multiple targets.
- Identifying common mechanisms of action from neuroactive compounds that shift the excitatory/inhibitory balance in synapses could guide repurposing efforts for a broad range of neurodevelopmental disorders.

**Abstract:**

**Background**: Neurodevelopmental disorders span a wide spectrum of deficits, often with a known or suspected genetic basis. While some genetic determinants may indicate treatment with selective compounds, more often both the molecular cause of the disorder and the mechanism of action for the therapeutic compound are more ambiguously matched. Due to the polypharmacological nature of most neuroactive compounds, measuring gene expression changes following drug perturbation could be an effective strategy to gain insight into shared therapeutic action downstream of diversity in receptor interaction. High-throughput drug discovery platforms have effectively measured changes in gene expression following drug perturbation in cell cultures, but unfortunately, these platforms often lack specificity for neuroactive compounds, fail to capture the developmental influence of cell–cell interactions, and do not accurately model drug metabolism in an intact system. **Methods**: In this study, we present a high-throughput, low-cost and cell-type-specific approach for capturing transcriptional changes in neural cell populations following neuroactive compound exposure through the combined use of transgenic zebrafish, cell sorting, and bulk RNA-seq. **Results**: Our system captures unique transcriptional profiles between neuronal and non-neuronal cell populations and demonstrates specific drug responsiveness within our neuronal cell population. We assessed two known positive allosteric modulators (PAMs) of γ-Aminobutyric acid sub-type A receptors (GABA_A_R), ivermectin and propofol, as a case study to explore shared pathway and gene expression changes following drug exposure; these chemically distinct agents share a mechanistic signature that dampens the neuronal hyperexcitability characteristic of a broad spectrum of neurodevelopmental disorders. Two shared downregulated genes reflect a core expression module for modulating GABAergic tone: SRC proto-oncogene, non-receptor tyrosine kinase (*SRC*), and Glutamate decarboxylase 2 (*GAD2*). **Conclusions**: We provide this methodology and analysis as a framework for exploring shared changes in gene expression following neuroactive compound exposure in vivo, leading to a more complete and nuanced understanding of therapeutic effects on neurons that can aid in drug repurposing efforts for neurodevelopmental disorders.

## 1. Introduction

### 1.1. Repurposing Neuroactive Compounds for Neurodevelopmental Disorders

Neurodevelopmental disorders represent a spectrum of heterogeneous disorders arising from genetic variation in thousands of known genes, as well as other undisclosed sources [1]. The diversity and complexity of the underlying causes makes a systematic approach to therapeutics challenging; despite the availability of compound libraries containing tens of thousands of drugs, the US FDA has approved only 1393 orphan drug designations across all rare disorders, and less than 100 are identifiable for the treatment of neurodevelopmental disorders [2,3]. However, modern technology has introduced useful tools to accelerate drug discovery based on common phenotypic and molecular underpinnings for these disorders. Automation for high-throughput screening in cell cultures has enabled the development of massive databases, like the LINCS 1000 Connectivity Map, which details drug responses fine-tuned to specific genetic perturbations [4]. Video and image processing for deep phenotyping has enabled rapid behavioral assessments of neuroactive compounds in model organisms, especially the zebrafish *Danio rerio* [5,6,7,8]. Additionally, the AI-aided design of novel drug compounds has supported ever-larger scale efforts to prioritize candidate therapeutics [9,10]. However, assessing the safety and efficacy of novel compounds remains a slow and intensive process. Drug repurposing aims to reposition drugs with known safety and pharmacokinetic profiles for new uses.

The potential for rapid deployment using a repurposed compound is especially attractive for disorders with rapidly growing prevalence, like Alzheimer disease, though results for neurodegenerative disorders have thus far been limited [11,12]. Despite the heterogeneity of neurodevelopmental disorders, drug repurposing screens have successfully led to new uses that manage complex symptoms. For example, drugs like the mTOR inhibitors everolimus and sirolimus have been used to control cell growth in tuberous sclerosis complex disorders, suggesting a common therapeutic approach for a broader spectrum of potential neurodevelopmental mTORopathies [13,14]. Gabapentin represents a further example of drug repositioning. This drug is increasingly used for pain management to fill the void left by reduced opioid prescribing [15]. While there is concern that the overuse of gabapentin may lead to similar drug abuses, its off-label use for numerous neurological and psychiatric conditions suggests a mechanism for beneficial neuromodulator activity [16]. The putative mechanism of action for gabapentin decreases excitatory neurotransmitter release, thereby dampening the excitatory/inhibitory (E/I) ratio proposed to be a core source of hyperexcitability across neurodevelopmental and neurodegenerative disorders [17,18,19]. This E/I nexus presents another attractive therapeutic hub for repurposing other compounds that normalize the E/I ratio, though understanding the cellular and genetic context for modulating either the excitatory or inhibitory contributions remains an active area of investigation [20,21].

### 1.2. GABAergic Neuroactive Compounds

Neuroactive compounds exert effects within the nervous system primarily through drug–receptor interactions. Despite their use to treat both neurological and psychiatric disorders, little attention has gone toward understanding what biological effects these compounds exert outside of interactions with their target receptors. This is in stark contrast to how they are identified: by their effects in behavioral assays long before their biological mechanisms of action are understood [22]. Additionally, most clinically effective neuroactive compounds interact with numerous receptor systems, making it difficult to gain insight into how cell–cell interactions and polypharmacology contribute to their therapeutic effects. By measuring changes in the intermediate biological signals induced by neuroactive compound exposure, it may be possible to gain insight into shared therapeutic mechanisms. Through the classification of compounds by shared changes in gene expression rather than known target receptor interaction, novel neuroactive compounds from non-traditional drug classes can be explored for broader therapeutic use through drug repurposing. In our study, we explore two chemically distinct positive allosteric modulators (PAMs) of γ-Aminobutyric acid sub-type A receptors (GABA_A_R), ivermectin and propofol, that serve to agonize the inhibitory component of the E/I ratio, providing a complementary approach to the proposed normalizing influence of gabapentin.

Rapid drug repurposing efforts were amplified during the recent COVID-19 pandemic, highlighting some of the opportunities and challenges for using known, safe drugs in new contexts. Ivermectin emerged from SARS-CoV-2 viral replication screens as an attractive candidate therapeutic to prevent COVID-19, given its known safety profile for treating parasitic worms at low doses. The mechanism underlying ivermectin effectiveness against parasites is due to its binding to glutamate-gated chloride channels at the worm neuromuscular junction, which induces paralysis and death. However, ivermectin can be neurotoxic in hosts, depending on dose, infection status, and genetics [23,24]. Further complicating matters, ivermectin has known polypharmacological effects in mammalian brain tissue: at low concentrations, these include putative PAM action on GABA_A_R, the main inhibitory receptor type in the brain; at higher concentrations, these include PAM action on inhibitory glycine receptors and nicotinic acetylcholine receptors as well [25,26]. The mechanism of action on GABA_A_R itself is also variable and has been shown to include full agonist activity at α_1_β_3_ receptors with a similar, though less potent, efficacy as GABA itself [27].

Anesthetics are another large class of commonly used neuroactive compounds with unclear polypharmacological mechanisms. Current theories suggest that complex interactions between different brain regions regulate the loss of consciousness under anesthesia. At a molecular level, anesthetics can be classified by their ability to either augment inhibitory receptors or antagonize excitatory receptors [28]. Propofol, a commonly used anesthetic, acts as a PAM of GABA_A_R [29,30]. In addition to GABA_A_R, propofol acts as a PAM of inhibitory glycine receptors while also inhibiting excitatory nicotinic acetylcholine receptors [31,32]. Propofol allosteric binding with α_1_β_3_ GABA_A_ receptors has been observed at three distinct sites [33].

Despite differing fundamentally in size, complexity, structural class, and clinical application, ivermectin and propofol share a complement of receptor targets that makes them a good pilot for assessing shared and divergent gene expression changes in drug-exposed neural cell populations.

### 1.3. Advantages for Drug Repurposing from Ex Vivo Neurons

To achieve high-throughput screening for compounds in particular cell populations, projects like LINCS 1000 rely on immortalized cell lines in 2D culture systems. Unfortunately, most of these cell lines are non-neuronal. One way to circumvent the lack of neuron-focused molecular signatures, which has been used to define Autism Spectrum Disorder (ASD) expression modules, derives the gene signatures from human clinical samples first and then compares them against the whole complement of drug profiles in the LINCS 1000 database [34]. This clever approach circumvents the limited availability of neuronal cell types, but allows for the influence and off-target interaction of compounds with cell types from other tissues. Furthermore, even among the available neuronal cell lines in the database, they often display characteristics that differ from primary neuronal cell lines [35]. In other contexts, primary cell lines themselves have been used for compound screens, though the outcomes remained focused on the phenotypic quantification of drug response [36]. Combined, these factors display the need for a low-cost, high-throughput model system to accurately capture biological signatures from primary neurons following neuroactive compound exposure.

Zebrafish are a low-cost model organism with promising applications and a long history in drug screening [37]. A single breeding pair of zebrafish can produce hundreds of genetically similar offspring, allowing for more robust sample sizes compared to other models. Additionally, compounds can be administered directly to embryonic zebrafish through bath immersion, where they are absorbed through the skin, digestive tract, or gills [38,39]. The nervous system develops rapidly and animals display controlled swimming behavior and touch-evoked startle response within as little as 2 days post-fertilization (dpf) [40]. The ability to assess neurologic behavior regularly and quickly in zebrafish has made them a desirable model for drug screening and deep phenotyping [41,42]. Due to the frequent use of zebrafish as a model for neurodevelopment, several transgenic lines expressing protein markers in neural cell populations are available as well [43].

In this study, we make use of transgenic lines to sort neuronal and non-neuronal drug-exposed cell populations based on their GFP expression using flow cytometry. This approach allows us to capture cell-type-specific transcriptional changes using RNA-seq. We present an analysis of GABA-acting compounds as a case study to explore shared transcriptional perturbations available from our approach.

## 2. Materials and Methods

### 2.1. Animals

A previously generated line of transgenic zebrafish on the AB background that expresses green fluorescent protein in response to neuroD, a neuronal transcription factor, was employed in this study, referred to as Tg(nrd:egfp) [43]. A single male and female pair of Tg(nrd:egfp) zebrafish were bred to produce embryos that were sorted into 6-well plates containing standard egg water before treatment.

### 2.2. Drug Dosing

Tg(nrd:egfp) embryos were exposed to select compounds from common drug classes. The compounds were dissolved in a chosen solvent before administration directly to embryo-containing egg water. Drug dosing was selected based on maximally tolerated doses established as part of a larger pilot study on assessing neuroactive compounds in *Danio rerio* (see Data Availability Section). Both propofol and ivermectin (Cayman Chemical, Ann Arbor, MI, USA) were prepared in <1% methanol solvent: propofol at 1 µg/mL and ivermectin at 1 µM. For each compound, biological triplicates of a pooled sample of 40 embryos from the same clutch were exposed at 1dpf for 24 h, consistent with the intermediate drug exposure time used in the LINCS1000 project [4].

### 2.3. Harvest and Cell Sorting

Following drug exposure, each pool of 40 embryos were homogenized to create single cell suspensions according to previously established protocols [44]. Suspensions with 1 µg/mL DAPI added were sorted using a BD FACSMelody 3-laser cell sorter in purity mode (BD Biosciences, Franklin Lakes, NJ, USA). DAPI+ dead cells were excluded and live cells were sorted by GFP+ expression into GFP+ and GFP−-sorted Tg(nrd:egfp) suspensions for each sample; the gating decision tree is provided in Supplementary Figure S1 and FCS files are provided in Supplementary Folder S1. Cell pellets were spun down and flash-frozen in liquid nitrogen. The number of sorted cells per sample is summarized in Supplementary Table S1; an average of <300,000 cells per sample dictate an ultra-low RNA sequencing approach.

### 2.4. Bulk RNA-Seq

Flash-frozen cell pellets were shipped to GeneWiz for RNA extraction, library preparation, and 150 bp paired-end sequencing on the Illumina HiSeq platform. RNA quality reports are summarized in Supplementary Table S1; all sample input was too low in concentration to be detected by TapeStation for RIN assessment, but visual inspection of the electropherograms shows distinct 18S/28S peaks indicative of undegraded, low-quantity samples with appropriate quality for library preparation. Library size distribution also follows the expected profile for a range of fragments at 300–500 bp with an average peak centered at 400 bp.

### 2.5. RNA-Seq Analysis

**Figure 1:**
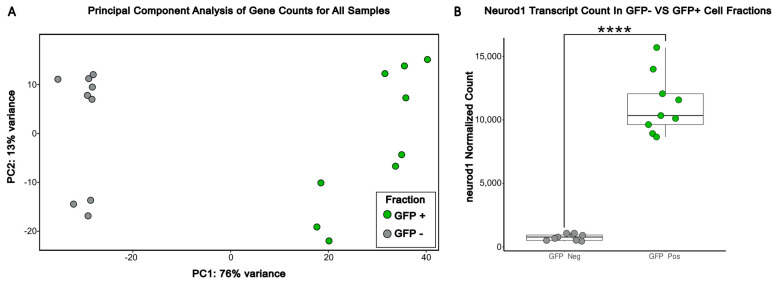
Principal component analysis displays successful separation of GFP+ and GFP− cell fractions by PC1 variance in the transcriptional profiles. (**A**) Principal component analysis was performed across ivermectin-, propofol-, and solvent-treated GFP+ and GFP− samples using a blind, variance-stabilized transformation of counts from DESeq2. (**B**) Neurod1 transcript expression was generated from normalized DESeq2 counts across all samples. Boxplots show the median (center line), interquartile range (box), and whiskers (1.5× interquartile range). Significance was determined via DESeq2-adjusted *p*-value (**** *p* < 0.0001).

**Figure 2:**
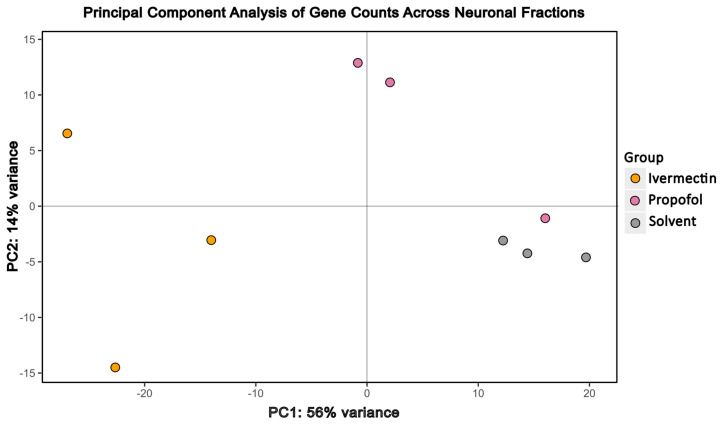
Principal component analysis of GFP+ samples alone reveals drug-specific effects. Principal component analysis was performed across neuronal fractions (GFP+) from ivermectin-, propofol-, and solvent-treated samples using a blind variance stabilized transformation of counts from DESEQ.

**Figure 3:**
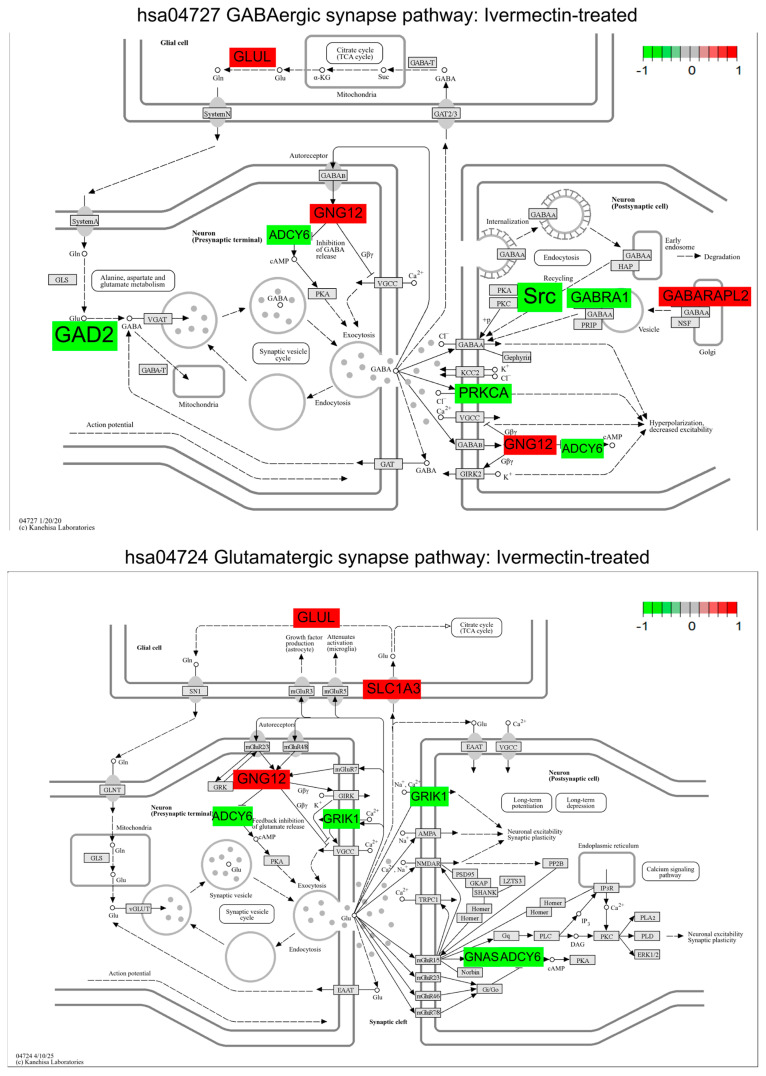
Ivermectin-treated neuronal cell populations display a downregulation in the KEGG GABAergic synapse (hsa04727) and glutamatergic synapse (hsa04724) pathways. GABAergic and glutamatergic synapse pathways were plotted with Pathview to display DEGs (*p*_adj_ < 0.1). Color shading is representative of the log_2_ (fold change) value for each DEG when comparing ivermectin treated neuronal fractions to the solvent control; green represents downregulation and red represents upregulation. Gene list and KEGG pathway labels vary: in the original KEGG pathway, *GAD2* is represented by GAD, *ADCY6* by AC, *GNG12* by Gi/o, *GABRA1* by GRIF, *GABARAPL2* by GABARAP, *GLUL* by GS/GLNS, *PRKCA* by GABA_C_, *SLC1A3* by EAAT, *GRIK1* by KA, and *GNAS* by G_S_; pathway image labels have been altered for legibility and to match the gene names in Table 1 and Table 2.

**Figure 4:**
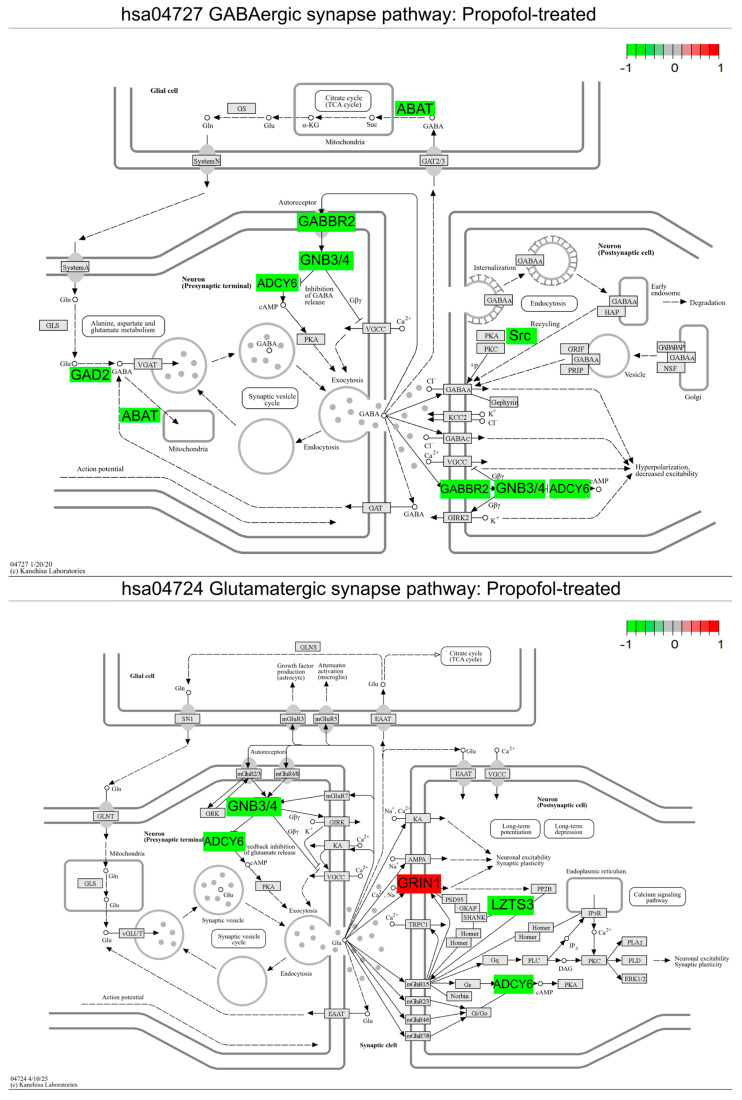
Propofol-treated neuronal cell populations display a downregulation in the KEGG GABAergic synapse (hsa04727) and glutamatergic synapse (hsa04724) pathways. GABAergic and glutamatergic synapse pathways were plotted with Pathview to display DEGs (*p*_adj_ < 0.1). Color shading is representative of the log_2_ (fold change) value for each DEG when comparing propofol-treated neuronal fractions to the solvent-treated control; green represents downregulation and red represents upregulation. Gene list and KEGG pathway labels vary: in the original KEGG pathway, *ABAT* is represented by GABA-T, *GAD2* by GAD, *ADCY6* by AC, *GNB3/4* by Gi/o, *GABBR2* by GABA_B_, and *GRIN1* by NMDAR; pathway image labels have been altered for legibility and to match the gene names in Table 1 and Table 2.

**Figure 5:**
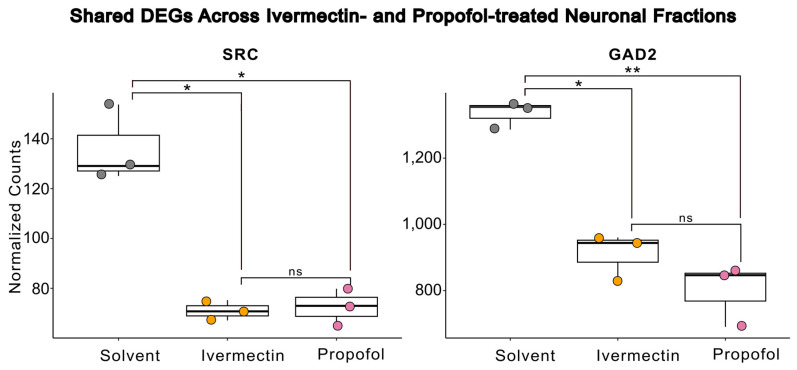
Ivermectin- and propofol-treated neuronal cell populations share DEGs. A significant downregulation in SRC proto-oncogene, non-receptor tyrosine kinase (*SRC*) and Glutamate decarboxylase 2 (*GAD2*) gene expression was observed in both ivermectin- and propofol-treated neuronal fractions compared to solvent-treated controls. Significance was determined via DESeq2 adjusted *p*-value (* *p* < 0.05, ** *p* < 0.01, ns = not significant).

Raw RNA-seq FASTQ files were aligned against the GRCz11.104 *Danio rerio* reference genome using Hisat2 (V 2.2.1) [45]. Count files were then generated based on exonal alignment using the HTSeq.scripts.count script in Python (V 3.7). Differential gene expression analysis was performed using DESeq2 (V 1.42.1) in R Studio (R V 4.3.3) [46]. DESeq objects were created by either loading in all conditions for across-drug comparisons (Figure 1 and Figure 2, Table 1 and Table 2) or only the specific drug of interest and solvent group (Figure 3 and Figure 4) before downstream analysis. When assessing shared regulation, the all-condition DESeq object is used again (Figure 5). Principal component analysis was performed using variance stabilized transformed counts following DESeq analysis. Zebrafish genes were mapped to the human gene space using gene homology mapping from a previously generated biomart query (accessed on January 2021) [47]. Homology-mapped DEGs lists were then filtered to retain only the lowest *p*-value in duplicate Entrez ID entries and null mappings were removed. Pathway analysis was performed on the filtered homology-mapped DEG list using Generally Applicable Geneset Enrichment (GAGE) (V 2.52.0) against a subset of the Kyoto Encyclopedia of Genes and Genomes (KEGG) (V 117.0, accessed on January 2021) specific for signaling and metabolic pathways using the sigmet.idx.hs index [48,49]. GAGE accommodates an experimental design with few replicates, and uses two-sample *t*-tests to model geneset-specific variance in a single list that combines both upregulated and downregulated genes expected to be coregulated in the same pathway. Both a *p*-value and a q-value (adjusted *p*-value, *p*_adj_) are reported; even with the benefits of GAGE, higher q-values are expected, especially for smaller datasets, and a cutoff of q ≤ 0.1 was used in method development [46]. We maintained a threshold of *p*_adj_ < 0.05 at the single gene level, relaxing the threshold to *p*_adj_ ≤ 0.2 as a means to maximally explore perturbation in pathways since some conditions have limited differential effects (small set sizes). GABAergic synapse and neuroactive ligand–receptor interaction pathways were visualized using Pathview (V 1.42.0) [50].

### 2.6. FDA Approval Search

Search of orphan drug designations and approvals (last accessed 5 February 2026) used default settings with a start date of 1 January 1983 and end date of 2 February 2026, and the field ‘Search Results’ was set to ‘Only Approved Products’ to determine all available, approved products. Refined searches updated the ‘Orphan Designation’ field to ‘*neurodevelopmental*’ OR ‘*neuro*’ OR ‘*syndrome*’.

## 3. Results

### 3.1. Flow-Sorting Tg(nrd:egfp) Zebrafish Embryos Captures Unique Transcriptional Profiles

We validated the successful capture of distinct cell populations via flow cytometry using a principal component analysis (PCA) across all samples. As expected, we observed clear separation of both cell fractions in principal component 1 (PC1) (Figure 1A). Furthermore, a significant difference in neuroD1 transcript counts between GFP+ and GFP− samples confirmed successful sorting (Figure 1B).

### 3.2. Principal Component Analysis Reveals Changes in Neuronal Cell Fractions Following Neuroactive Compound Exposure

Next, we sought to explore drug-specific effects among the enriched neuronal (GFP+) cell populations. Principal component analysis was conducted among the GFP+ cell fractions and PC1 captured the strongest transcriptional changes, clearly delineating the transcriptional profiles of different drug treatment conditions among neural cells (Figure 2). Ivermectin displayed the largest separation from the solvent control compared to propofol. Primary data for Figure 1 and Figure 2 is provided as Supplementary Table S1.

### 3.3. Gene Set Enrichment Analysis Demonstrates a Significant Downregulation in Gabaergic and Glutamatergic Synapse Pathways Across GABA-Acting Compounds

We then performed differential gene expression analysis on both ivermectin- and propofol-treated cell fractions to capture differentially expressed genes (DEGs) in the enriched neural cell populations compared to the solvent control. To determine relevant biological pathways from DEGs, gene set enrichment analysis was conducted using GAGE. The GABAergic synapse pathway was significantly downregulated in both ivermectin- and propofol-treated neuronal fractions compared to the control. Additionally, we observed that the glutamatergic synapse pathway was significantly downregulated across both treatments (Figure 3 and Figure 4). We considered whether these results could reflect a non-specific response to any compound exposure and chose a compound from the larger study, donepezil, a cholinesterase inhibitor, as a compound with limited expected direct effects on GABAergic and glutamatergic synapse maps. Repeating our analysis revealed that hsa04727 GABAergic synapse is not listed at any rank on either up- or downregulated lists after donepezil exposure. Likewise, hsa04724 for the glutamatergic synapse is absent from donepezil’s upregulated list and ranks poorly (rank 41) on the downregulated list (Supplementary Tables S3–S6). We then investigated DEGs in the GABAergic and glutamatergic pathways to gain insight into gene-specific changes across compounds (Figure 3 and Figure 4). Primary data for Figure 3 is provided as Supplementary Tables S7–S10. Primary data for Figure 4 is provided as Supplementary Tables S11–S14.

### 3.4. Pathway-Guided Differential Expression Analysis Identifies Shared DEGs Within the Gabaergic Synapse Pathway Across GABA-Acting Compounds

Due to our interest in shared transcriptional changes across GABA-A receptor-acting compounds, we focused our investigation on DEGs within the GABAergic synapse pathway. We compared GABA pathway-specific DEGs across ivermectin- and propofol-treated neuronal fractions to identify shared transcriptional differences. Glutamate decarboxylase 2 (*GAD2*) and SRC proto-oncogene, non-receptor tyrosine kinase (*SRC*), were identified as shared DEGs in both ivermectin- and propofol-treated neuronal fractions (Table 1) (Figure 5). Primary data for Figure 5 is provided as Supplementary Tables S15–S17. To contrast the GABAergic synapse pathway, we also identified shared DEGs within the glutamatergic synapse pathway, although none reached significance across either ivermectin- or propofol-treated neuronal fractions (Table 2).

## 4. Discussion

In this study, we show that enriched neuronal cell populations displaying unique transcriptional profiles can be captured using transgenic zebrafish lines and cell sorting. In addition, we provide an exploratory analysis of two GABA_A_R-acting compounds (ivermectin and propofol) and found that their target-defined drug class was captured by the identification of the GABAergic synapse pathway, which was significantly downregulated in both treatments. In addition, we identified specific DEGs in the GABAergic synapse pathways that are significantly dysregulated across both treatments.

Most dysregulated genes are involved in remodeling the GABAergic synapse; SRC was significantly downregulated in both drug exposure conditions, which was unexpected. However, there is a known E/I switch at GABAergic synapses at this stage in zebrafish development [51]. During the E/I switch, *slc12a5b* gene expression rises, leading to Cl export through KCC2b and converting GABA_A_R from an excitatory to inhibitory state. Detectable *slc12a5b* gene expression in all samples (Gene ID ENSDARG00000078187; Supplementary Tables S2 and S6) suggests that the regulated switch from excitatory to inhibitory GABAergic signaling had begun and was not dysregulated due to drug treatment, but the switch may not have been completed. Under either PAM or agonist treatment at the inhibitory synapse, *SRC* upregulation would be expected to 1) maintain GABA_A_R at the membrane through the downregulation of AP2 and reduced endocytosis [52], or 2) enhance GABA_A_R function through intracellular phosphorylation [53]. Our data, where both ivermectin and propofol equally suppress *SRC* expression, is most consistent with the idea that the downregulation of *SRC* may be a compensatory mechanism early in development in normal neurons exposed to PAMs of GABA_A_R in their excitable, rather than inhibitory, state.

In addition, *GAD2*, the enzyme responsible for converting glutamate to GABA, is significantly downregulated, suggesting direct transcriptional control over neurotransmitter production that limits GABA production in treated cells. While not statistically significant, ivermectin trends toward greater suppression of *GAD2*, which may be indicative of its potential agonistic action. Alternatively, propofol is known to have a rapid clinical metabolism in humans [54], which could explain the weaker transcriptional effects of propofol more broadly in our study. However, in investigations for propofol use as an anesthetic in adult zebrafish, even short, minutes-long exposures before recovery produced measurable deficits in animal activity 24 h later, suggesting some longer-term biological response to even low doses and short exposures [55]. Similar changes in membrane-associated activity at the synapse have also been observed in hippocampal neurons under low-dose propofol exposure in neonatal mice, further highlighting the potential for findings in zebrafish to translate to mammalian systems [56]. Intact neuronal circuitry is important to observe these effects, as expected synaptic remodeling is not observed in propofol-treated cancer cell cultures, where cell cycle effects driven by tumorigenesis dominate instead [57].

Together, these shared gene responses reveal the importance of identifying critical treatment windows for neurodevelopmental disorders, as well as how these windows may shift or switch effect due to context. For example, mutations in *SLC12A5* lead to an infantile epileptic syndrome that would be expected to respond differentially to E/I-directed GABAergic treatment strategies [58]. We look forward to expanding the assessed timepoints of this pilot study in the future, as well as introducing genetic perturbations to the zebrafish prior to performing the drug screening to better model the translational potential of this approach.

Aside from the shared gene expression, the GABA_A_R drugs in this pilot each influence the unique expression of both receptor and metabolic targets. Notably, propofol treatment results in (1) the downregulation of G-protein receptor subunits (e.g., *GNB3* and *GNB4*) and (2) the downregulation of *ABAT*, also called GABA-T, the rate-limiting enzyme responsible for breaking down GABA. The GNB gene family plays a well-established role in neurodevelopment, and a wide variety of genetic variants are associated with neurodevelopmental disorders, including the availability of zebrafish models for 3 family members including *GBN3* [59]. Without sufficient breakdown, GABA could accumulate in the synaptic cleft, consistent with the expected positive modulatory effects from propofol. Pharmacological inhibitors of the GABA-T enzyme have been established for the treatment of some infantile epileptic syndromes, providing a rationale for considering similar pharmacological approaches that may manage the primary clinical presentation while also allowing for diverse beneficial effects on secondary phenotypes [60].

GABAergic effects also extend to the regulation of gene expression at the opposing, excitatory glutamatergic synapses. While no common genes are significantly differentially expressed in both conditions, the fold changes are larger and affect unique genes, reflecting a more diverse and divergent mechanism of action on adjacent glutamatergic processes. We hypothesize that genes involved in ivermectin response would dampen synaptic excitability through the control of excitatory neurotransmitter persistence: downregulating GRIK1 ionotrophic receptors for glutamate while upregulating clearance of the transmitter through the SLC1A2 glutamate transporter. Conversely, we hypothesize that propofol acts more directly on canonical glutamatergic signaling through NMDA receptors, while further remodeling the synapse to downregulate G-protein receptor cascades. Neither of these responses are typically reported activities; they represent an expanded action for each compound that can be tested through future functional investigation. The modulatory and atypical signaling responses have been appreciated in general for a while, especially considering that behavioral outputs in human patients sometimes directly conflict under the same treatment regimens [61], and it is much more difficult to capture the biological context directing these differential phenotypic responses. Combining our gene expression studies with additional biological readouts at additional developmental timepoints will help us understand the variations in drug response across multiple signaling hubs.

This study provides a foundation using cell sorting in transgenic zebrafish to capture transcriptional changes following drug perturbation in enriched neuronal cell populations. The approach is lower in cost and uses more accessible instrumentation and bioinformatics workflows than the scRNA-seq technologies that would supplant this approach in the future. Furthermore, our approach provides an additional opportunity to assess in vivo neuronal phenotyping from initial exposure until sequencing in the same organism. Using zebrafish as a model also opens up the possibility for future studies at the intersection of genetic perturbation, using CRISPR-Cas9 gene editing or similar technologies, and drug effects in vivo. By testing candidate compounds across multiple target drug classes, we hope to provide specific transcriptional profiles that act as a shared mechanistic nexus leading to opportunities for future drug repurposing.

## 5. Conclusions

This combined method using transgenic GFP expression in neurons and a simple FACS procedure to collect small populations of target cells enables us to rapidly extract transcriptional signatures directly from intact primary neurons. The simplicity and speed of the procedure enables medium-throughput drug screening to assess downstream, molecular responses to compound exposure. The ability to assess transcriptional change in endogenous cell populations, especially neurons, expands on data from cultured cell databases such as the Connectivity Map, which provides a translational scaffold for repurposing candidate drugs. Here, chemically distinct GABA-acting drugs produce shared transcriptional signatures in zebrafish neurons that influence both GABAergic and glutamatergic synapses, which can be used to craft further functional studies of coordinated signaling hubs.

## Figures and Tables

**Table 1:** GABA-acting compounds ivermectin and propofol differentially downregulate expressed genes in the GABAergic synapse pathway (KEGG pathway map hsa04727).

Gene Name	Gene Symbol	*p*_adj_	log_2_ (Fold Change)
Ivermectin			
glutamate–ammonia ligase	*GLUL*	0.003	0.78
G protein subunit gamma 12	*GNG12*	0.007	2.6
GABA type A receptor associated protein like 2	*GABARAPL2*	0.017	0.84
**SRC proto-oncogene, non-receptor tyrosine kinase**	***SRC***	**0.017**	**−0.94**
trafficking kinesin protein 2	*TRAK2*	0.021	−0.83
**glutamate decarboxylase 2**	***GAD2***	**0.024**	**−0.55**
gamma-aminobutyric acid type A receptor subunit alpha1	*GABRA1*	0.052	−0.73
adenylate cyclase 6	*ADCY6*	0.057	−0.93
G protein subunit beta 4	*GNB4*	0.064	−0.66
protein kinase C alpha	*PRKCA*	0.083	1.89
Propofol			
4-aminobutyrate aminotransferase	*ABAT*	0.001	−1.46
**glutamate decarboxylase 2**	***GAD2***	**0.002**	**−0.74**
G protein subunit beta 3	*GNB3*	0.014	−1.65
G protein subunit beta 4	*GNB4*	0.019	−0.86
adenylate cyclase 6	*ADCY6*	0.024	−1.15
**SRC proto-oncogene, non-receptor tyrosine kinase**	***SRC***	**0.043**	**−0.91**
glutamate–ammonia ligase	*GLUL*	0.06	−2.02
calcium voltage-gated channel subunit alpha1 F	*CACNA1F*	0.082	−2.17
gamma-aminobutyric acid type B receptor subunit 2	*GABBR2*	0.098	−1.45

Rows with downregulated genes common to ivermectin and propofol treatment at *p*_adj_ < 0.1 are shown in bold.

**Table 2:** GABA agonist compounds ivermectin and propofol differentially downregulate expressed genes in the glutamatergic synapse pathway (KEGG pathway map hsa04724).

Gene Name	Gene Symbol	*p*_adj_	log_2_ (Fold Change)
Ivermectin			
solute carrier family 1 member 3	*SLC1A3*	0	1.62
glutamate ionotropic receptor kainate type subunit 1	*GRIK1*	0.001	−2.47
glutamate–ammonia ligase	*GLUL*	0.003	0.78
GNAS complex locus	*GNAS*	0.004	−1.23
G protein subunit gamma 12	*GNG12*	0.007	2.6
solute carrier family 1 member 7	*SLC1A7*	0.05	−3.57
adenylate cyclase 6	*ADCY6*	0.057	−0.93
G protein subunit beta 4	*GNB4*	0.064	−0.66
protein kinase C alpha	*PRKCA*	0.083	1.89
solute carrier family 1 member 1	*SLC1A1*	0.086	0.76
Propofol			
G protein subunit beta 3	*GNB3*	0.014	−1.65
G protein subunit beta 4	*GNB4*	0.019	−0.86
adenylate cyclase 6	*ADCY6*	0.024	−1.15
glutamate ionotropic receptor NMDA type subunit 1	*GRIN1*	0.034	0.86
leucine zipper tumor suppressor family member 3	*LZTS3*	0.039	−1.11
glutamate–ammonia ligase	*GLUL*	0.06	−2.02

## Data Availability

The original data presented in the study are openly available in Zenodo (https://doi.org/10.5281/zenodo.18372315).

## Supplementary Materials

The following supporting information can be downloaded at https://www.mdpi.com/article/10.3390/brainsci16030323/s1: A readme file describing all Supplementary Material. Files S1–S8: All code used in main figure, table, and corresponding supplementary table generation is available as .Rmd files. Table S1: Method supplement for QC. Tables S2–S17: Corresponding tables to the R analysis are provided for reference (S2: Figure 1 and Figure 2; S3–S6: Figure 3 and Figure 4; S7–S10: Figure 3; S11–S14: Figure 4; S15–S17: Figure 5). Figure S1 and Folder S1: Flow cytometry method support and primary data files. Figures S2 and S3: Volcano plots for ivermectin and propofol.

## Abbreviations

The following abbreviations are used in this manuscript:

PAM	Positive allosteric modulators
GABA_A_R	γ-Aminobutyric acid sub-type A receptor
E/I	Excitatory/Inhibitory
PCA	Principal component analysis
ASD	Autism Spectrum Disorder
