# Supplementary material for: Neuro-Transcriptomic Responses to Polypharmacological Agents in Danio rerio: Implications for Translational Drug Repurposing in Neurodevelopmental Disorders
Source: Brain Sci. 2026 Mar 18;16(3):323. doi: 10.3390/brainsci16030323 (PMC13025160; doi:10.3390/brainsci16030323)
Supplement: Supplementary file 1 [file brainsci-16-00323-s001.zip › README.pdf]

Methods supplements

Supplementary_Figure_S1.png

Decision Tree for Flow Cytometry

Supplementary_Folder_S1

Flow Cytometry FCS files for all samples plus instrument control

Supplementary_Table_S1.xlsx

Summary-QC-metrics

Code Files for each figure, file name includes the supplements that were generated from each code file in addition:

Supplementary_File_S1.Rmd

R_Code_Figure_1_and_Supplementary_Table_1

Supplementary_File_S2.Rmd

R_Code_Figure_2

Supplementary_File_S3.Rmd

R_Code_Figure_3_and_Supplementary_Table_2_3_4_5

Supplementary_File_S4.Rmd

R_Code_Figure_4_and_Supplementary_Table_6_7_8_9

Supplementary_File_S5.Rmd

R_Code_Figure_5_and_Supplemental_Table_10_11_12.Rmd

Supplementary_File_S6.Rmd

R_Code_Table_1

Supplementary_File_S7.Rmd

R_Code_Table_2

Biomart supplementary file used for human homology:

Supplementary_File_S8.txt

martquery_1229190344_748 (BioMart query file)

Figure 1/2 Supplementary Table (zebrafish DEGs (GFP+ vs GFP- cells) used for NEUROD comparison and PCA generation):

Supplementary_Table_S2.csv

IVER_PROP_MEOH_POSvsNEG_zebrafish

Supplementary_Table_S3.csv

DNPZ_POS_VS_MEOH_POS_zebrafish

Supplementary_Table_S4.csv

DNPZ_POS_VS_MEOH_POS_human_filtered

Supplementary_Table_S5.csv

DNPZ_POS_VS_MEOH_POS_human_kegg_Upregulated_pathways_TOP_100

Supplementary_Table_S6.csv

DNPZ_POS_VS_MEOH_POS_human_kegg_downregulated_pathways_TOP_100

Figure 3 supplementary tables, (zebrafish DEGS, Human homology DEGS, Kegg Upregulated and Downregulated Pathways):

Supplementary_Table_S7.csv

IVER_POS_VS_MEOH_POS_zebrafish

Supplementary_Table_S8.csv

IVER_POS_VS_MEOH_POS_human_filtered

Supplementary_Table_S9.csv

IVER_POS_VS_MEOH_POS_human_kegg_Upregulated_pathways_TOP_100

Supplementary_Table_S10.csv

IVER_POS_VS_MEOH_POS_human_kegg_downregulated_pathways_TOP_100

Figure 4 supplementary tables, (zebrafish DEGS, Human homology DEGS, Kegg Upregulated and Downregulated Pathways):

Supplementary_Table_S11.csv

PROP_POS_VS_MEOH_POS_zebrafish

Supplementary_Table_S12.csv

PROP_POS_VS_MEOH_POS_human_filtered

Supplementary_Table_S13.csv

PROP_POS_VS_MEOH_POS_human_kegg_Upregulated_pathways_TOP_100

Supplementary_Table_S14.csv

PROP_POS_VS_MEOH_POS_human_kegg_downregulated_pathways_TOP_100

Figure 5 supplementary tables, (all zebrafish DEGS used for comparison/statistics):

Supplementary_Table_S15.csv

IVERPOS_vs_MEOHpos_zebrafish

Supplementary_Table_S16

PROPPOS_vs_MEOHpos_zebrafish

Supplementary_Table_S17.csv

IVERPOS_vs_PROPpos_zebrafish
