# Supplementary figures and images for: Neuro-Transcriptomic Responses to Polypharmacological Agents in Danio rerio: Implications for Translational Drug Repurposing in Neurodevelopmental Disorders
Source: Brain Sci. 2026 Mar 18;16(3):323. doi: 10.3390/brainsci16030323 (PMC13025160; doi:10.3390/brainsci16030323)

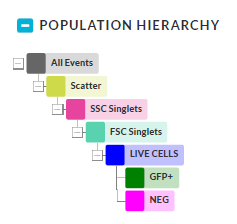

Supplement: Supplementary file 1 [file brainsci-16-00323-s001.zip › Supplementary_Figure_S1.png]

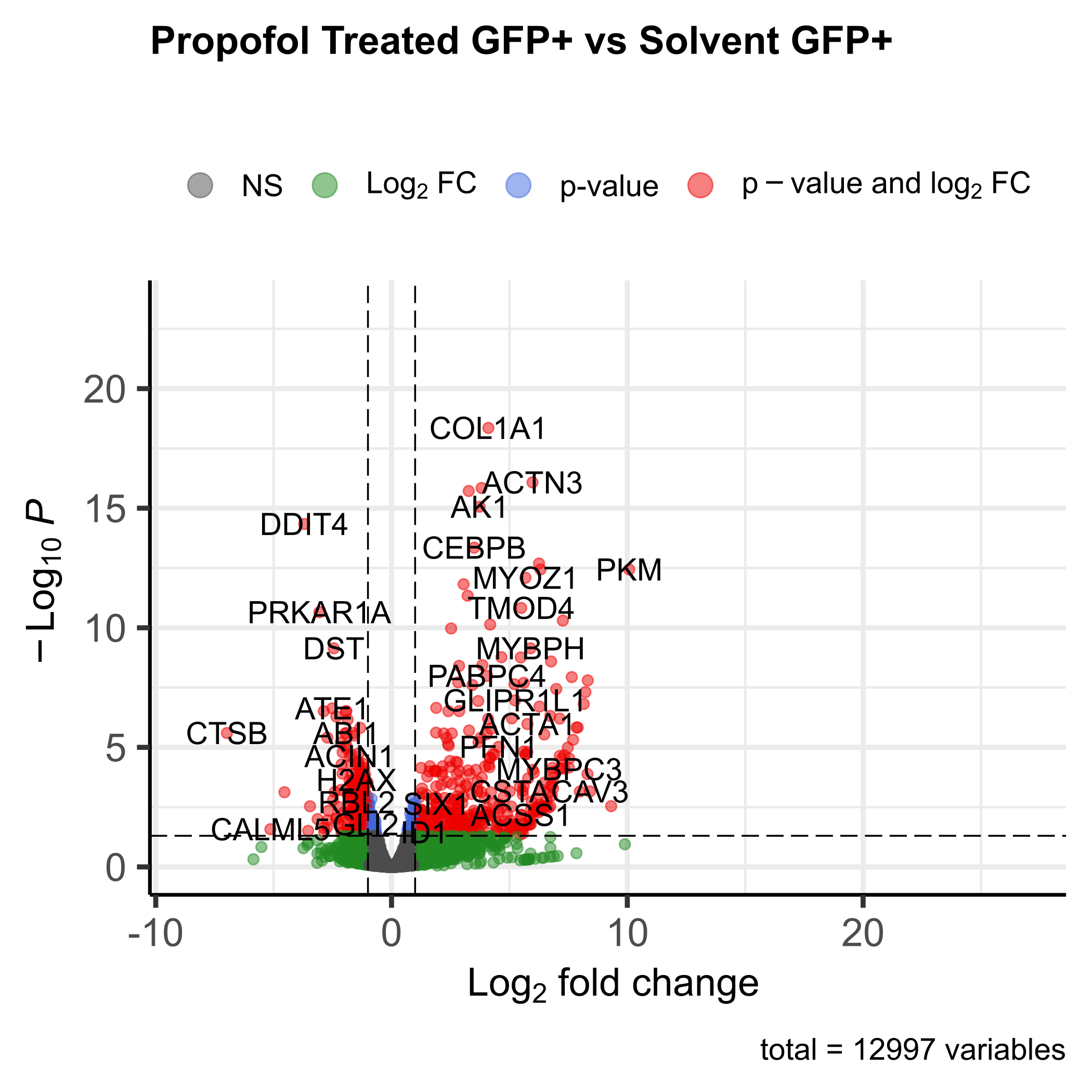

Supplement: Supplementary file 1 [file brainsci-16-00323-s001.zip › Supplementary_Figure_S2.png]

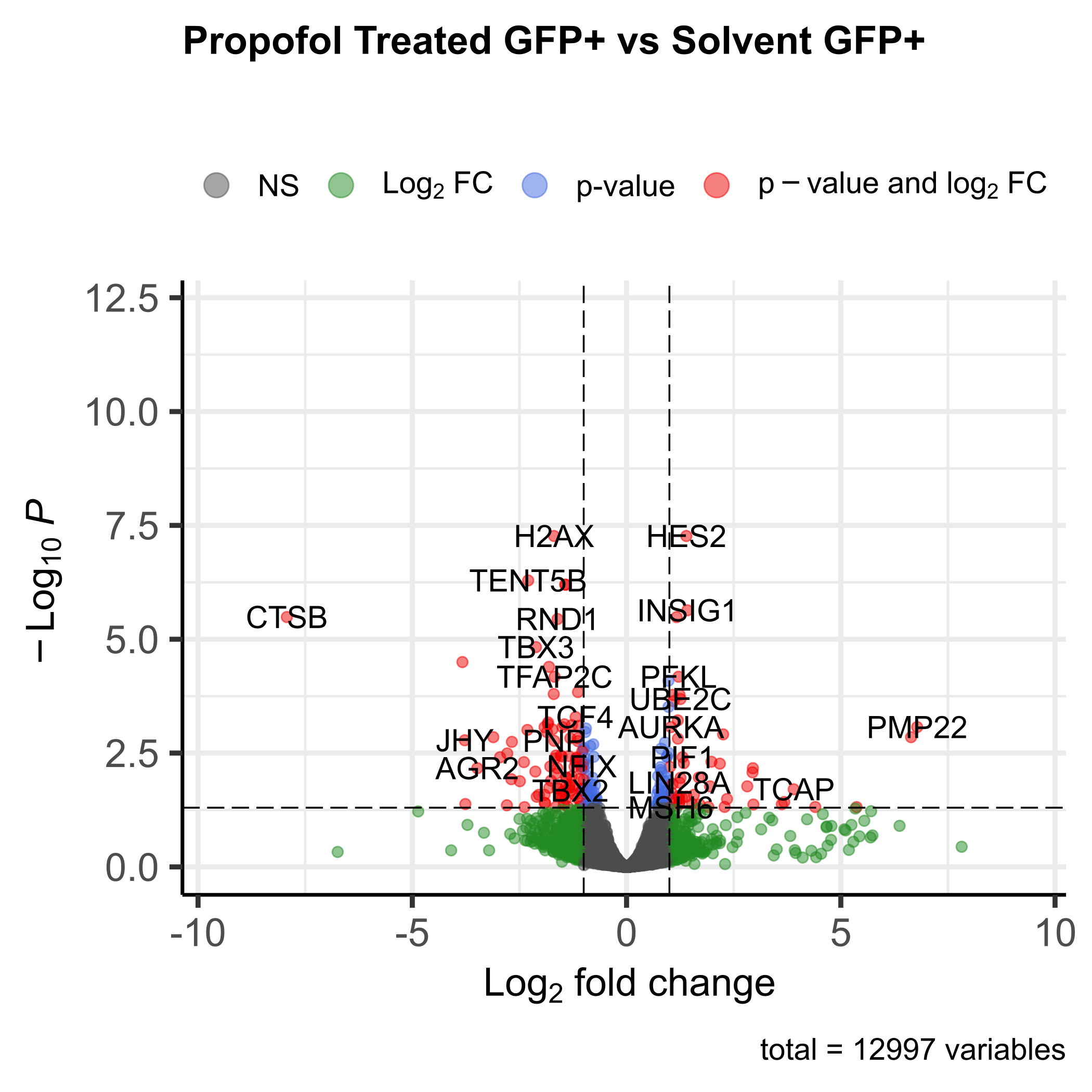

Supplement: Supplementary file 1 [file brainsci-16-00323-s001.zip › Supplementary_Figure_S3.png]
